# The Herb-Drug Pharmacokinetic Interaction of Fluoxetine and Its Metabolite Norfluoxetine with a Traditional Chinese Medicine in Rats by LC-MS/MS

**DOI:** 10.1155/2019/2471870

**Published:** 2019-11-30

**Authors:** Lijing Yan, Sheng Wang, Linlin Zhao, Juan Qiu, Lu Zhou, Wenbo Wang, Xia Xu, Dongsheng Wang, Xinjian Qiu, Dilan Qin

**Affiliations:** ^1^Laboratory of Ethnopharmacology, Institute of Integrated Traditional Chinese and Western Medicine, Xiangya Hospital, Central South University, Changsha, Hunan 410008, China; ^2^The Second Clinical Medical College, Zhejiang Chinese Medicine University, Hangzhou, Zhejiang 310053, China; ^3^Department of Health Management, The Third Xiangya Hospital of Central South University, Changsha, Hunan 410013, China; ^4^Department of Nuclear Medicine, The Third Xiangya Hospital of Central South University, Changsha, Hunan 410008, China; ^5^Environmental Monitoring Centre of Hunan Province, State Environmental Protection Key Laboratory of Monitoring for Heavy Metal Pollutants, Changsha, Hunan 410019, China

## Abstract

**Background:**

Fluoxetine (FLU) is the first-line and widely used medication for depression. The combination of Chaihu Shugan san (CSGS) and FLU is commonly used to enhance antidepressant effects and reduce side effects.

**Objective:**

The primary objective of this study was to investigate the potential pharmacokinetic effect of CSGS on FLU.

**Materials and Methods:**

Thirty-two healthy adult male Sprague-Dawley (SD) rats were randomly divided into four groups, the fluoxetine group and multiple dose groups A, B, and C. The rats in the different groups were orally administered with a combination of FLU and different doses of CSGS for 14 d. On the fifteenth day, serial blood samples were taken from the caudal vein before the administration and at 0.25, 0.5, 0.75, 1, 2, 4, 6, 8, 10, 12, 24, 36, and 48 h after the administration. A liquid-liquid extraction method was applied to extract the analytes from serum. Then, the concentrations of FLU and its metabolite, norfluoxetine (NOF), were determined using liquid chromatography-tandem mass spectrometry (LC-MS/MS). The pharmacokinetic parameters were calculated by DAS 3.2.8 program and compared by statistic analysis.

**Results:**

Compared with the FLU group, the FLU and NOF area under the plasma concentration-time curve (AUC) (0–∞) in multiple dose group C was significantly increased, while the NOF AUCs (0–∞) in multiple dose group A and multiple dose group B were decreased. Compared with the FLU group, the NOF clearance (CL) in multiple dose group C was decreased, while the CL in multiple dose groups A and B was increased.

**Discussion and Conclusion:**

There were some differences in pharmacokinetic parameters between the FLU group and multiple dose groups, and CSGS can affect the pharmacokinetics of fluoxetine.

## 1. Introduction

Depression is a mood disorder characterized by persistent feeling of sadness, loss of interest, decline in thinking and cognitive function, and disorder of physiological function [1]. According to the World Health Organization (WHO), there will be more than 300 million depression patients worldwide by 2020 [2], and depression could be the third principal cause of disability worldwide [3]. In addition, depression can easily lead to suicide and decreased fertility [4–6]. Based on data from the 2012 China Family Panel Studies survey, studies have shown that mental illness contributes to 14.7% of total personal expected medical spending in China, with depression and depressive symptoms accounting for 6.9% and 7.8%, respectively [7]. Therefore, it is not difficult to conclude that depression is a major neurological disease that poses a serious threat to human health and quality of life globally. Meanwhile, the related costs will cause a heavy economic burden to the society and family, and there is an urgent need for safe and effective treatment options [8].

Fluoxetine is a typical serotonin reuptake inhibitor and a commonly used antidepressant in the clinic. All types of antidepressants are ineffective in 30%–40% of patients, and most of them have problems such as delayed efficacy, large side effects, and poor tolerance [9, 10]. Taking FLU for a long time can cause severe side effects such as fatigue, headache, loss of appetite, weight gain, nausea, and bad mood [11]. Objective or subjective serious side effects often lead some patients to abandon medication [12].

It is estimated that up to 80% of the population in developing countries use traditional herbs for primary health care [13]. The combination of Chaihu Shugan san (CSGS) and FLU is commonly used to enhance antidepressant effects and reduce side effects [14], which have been confirmed by numerous studies [15–17]. Whether there is drug interaction between the two drugs is unknown. DDI (drug-drug interaction) is defined as the process in which a drug changes the absorption, distribution, and metabolism of the other drug, when two drugs are taken together [18]. DDI is a main concern in adverse drug reactions [19]. How to conduct coadministration more reasonably and safely needs to attract our attention. Regulators, including the US Food and Drug Administration, the European Medicines Agency, and the Japanese Medicines and Medical Devices Administration, have requested drug recommendations and management strategies for patients in their DDI guidance documents [20].

The potential effect of CSGS on pharmacokinetics of FLU is still unknown. LC-MS/MS with high separation efficiency and sensitive detection is the main technology for screening and analyzing active components [21]. Therefore, an LC-MS/MS method for the estimation of FLU and NOF in plasma was developed and validated. The aim of this study was to evaluate the optimal plasma levels of FLU and NOF when FLU is combined with different concentrations of CSGS. As a result, we could adjust the doses of CSGS and further improve the antidepressant effect of the combination.

## 2. Results and Discussion

### 2.1. Method Validation

Table 1 lists the regression equation, correlation coefficients, and LOQ of the analytes. The regression coefficients (*r*) were all higher than 0.99, showing a good linear relationship. Generally, the S/N ratio of LOD is 3 and LOQ is 10. LOD of FLU and norfluoxetine is 0.0082 ng/ml and 0.017 ng/ml. LOQ of FLU and norfluoxetine is 0.0246 ng/ml and 0.0512 ng/ml.

**Figure 1:**
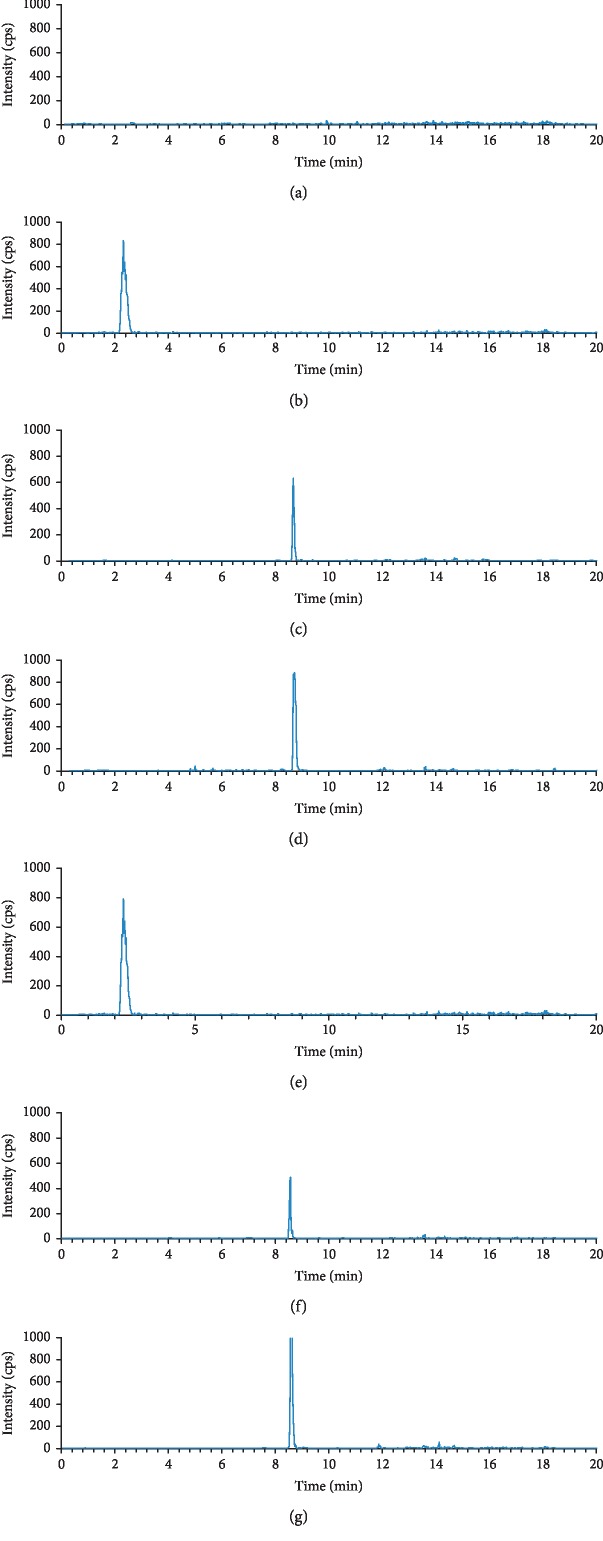
Chromatograms: (a) blank plasma; (b) blank plasma with IS; (c) blank plasma with fluoxetine; (d) blank plasma with norfluoxetine; (e) blood sample after oral administration with IS; (f) fluoxetine after oral administration; (g) norfluoxetine after oral administration. Chromatograms (e–g) are based on blood samples collected at 2 h after administration of a combination of FLU and high dose of CSGS (CSGS 23.6 g/kg·d, FLU 1.8 mg/kg·d). The retention times of IS, FLU, and NOF were 2.4, 8.5, and 8.6 min, respectively.

Figure 1 shows the typical chromatograms of blank plasma, plasma sample obtained from rats after oral administration, and blank plasma spiked with reference standards. Under the optimized condition, no endogenous interference was observed in the retention times of all tested matrices. The retention times of sulfamethoxazole (SMZ), FLU, and NOF were 2.4, 8.5, and 8.6 min, respectively.

Table 2 lists the intraday and interday precision and recovery of the analytes. RSD% of intraday variations for FLU and NOF was in the range of 2.35%–4.96% and 3.31%–4.20%, respectively. RSD% of interday precision for FLU and norfluoxetine was in the range of 2.70%–5.15% and 3.00%–4.34%, respectively. All the RSD% values were within the acceptable limit of <15%. Therefore, this method was suitable for the accurate quantification of rat biological samples.

Table 3 lists the stability of the analytes at three states. The data showed that RSD% values of the analytes at three states, at room temperature for 4 h, after three freeze-thaw cycles and at the storage temperature (−20°C) for 4 weeks, were within 10%, showing a good stability.

Tables 4 and 5 list the recoveries and matrix effect of the FLU and NOF. It could be seen that the recoveries of FLU and NOF (low, medium, and high concentrations) were in the range of 101.16%–104.06% and 92.31%–98.88%, respectively. RSD% of FLU and NOF was in the range of 2.19%–4.68% and 3.99%–6.20%, respectively. The matrix effects of FLU and NOF were within 100.18%–101.03% and 92.53%–97.91%, respectively, with RSD% less than 10%. Additionally, these results of recoveries and matrix effects also demonstrated that the method of liquid-liquid extraction was efficient and acceptable.

All above results proved that the newly developed LC-MS/MS method was sensitive, reliable, and enough for the simultaneous determination of FLU and NOF in rat plasma.

### 2.2. Pharmacokinetic Effect of the Herbal Drug with Fluoxetine in Rat Plasma

**Figure 2:**
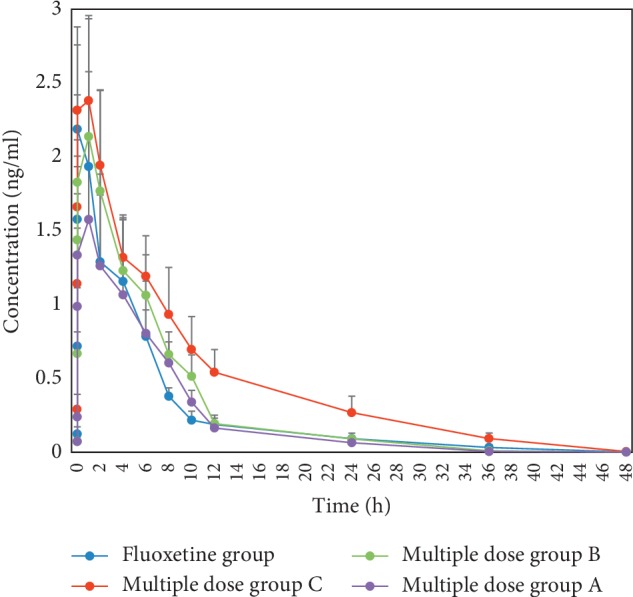
Concentration-time curves of fluoxetine (*n* = 8, mean ± SD). Fluoxetine group: fluoxetine 1.8 mg/kg·d for consecutive 14 d; multiple dose group A: CSGS 5.9 g/kg·d and fluoxetine 1.8 mg/kg·d for consecutive 14 d; multiple dose group B: CSGS 11.8 g/kg·d and fluoxetine 1.8 mg/kg·d for consecutive 14 d; multiple dose group C: CSGS 23.6 g/kg·d and fluoxetine 1.8 mg/kg·d for consecutive 14 d.

**Figure 3:**
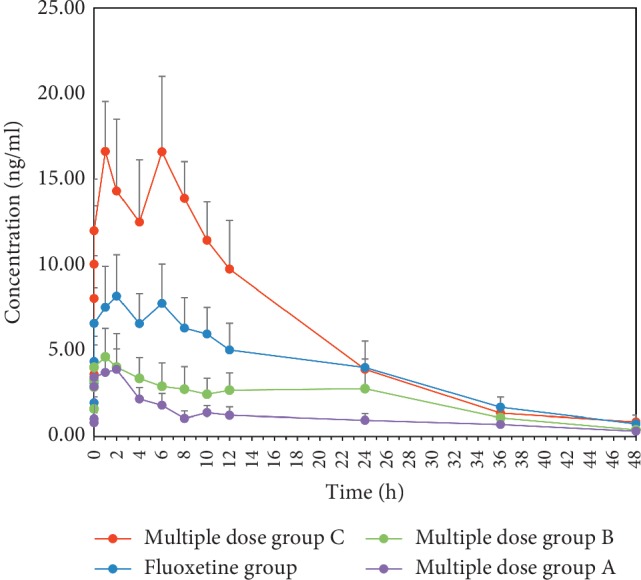
Concentration-time curves of norfluoxetine (*n* = 8, mean ± SD). Fluoxetine group: fluoxetine 1.8 mg/kg·d for consecutive 14 d; multiple dose group A: CSGS 5.9 g/kg·d and fluoxetine 1.8 mg/kg·d for consecutive 14 d; multiple dose group B: CSGS 11.8 g/kg·d and fluoxetine 1.8 mg/kg·d for consecutive 14 d; multiple dose group C: CSGS 23.6 g/kg·d and fluoxetine 1.8 mg/kg·d for consecutive 14 d.

The maximum plasma concentration (*C*_max_) and time to reach the maximum concentrations (*T*_max_) were obtained directly from the observed values illustrated in Figures 2 and 3. The pharmacokinetic parameters of FLU and NOF are listed in Tables 6 and 7.

The AUCs (0–∞) of FLU in FLU group, multiple dose group A, multiple dose group B, and multiple dose group C were 12.20 ± 2.67, 11.20 ± 3.86, 14.54 ± 3.22, 22.01 ± 4.49 *μ*g/L∗h, respectively. Compared to the fluoxetine group, the AUC (0–∞) of multiple dose group C was significantly increased. The AUCs (0–∞) of NOF in FLU group, multiple dose group A, multiple dose group B, and multiple dose group C were 181.93 ± 45.71, 53.27 ± 11.90, 101.74 ± 24.46, and 284.02 ± 49.07 *μ*g/L∗h, respectively. Compared to the fluoxetine group, the AUCs (0–∞) of norfluoxetine in multiple dose group A and multiple dose group B were decreased; however, multiple dose group C was the opposite.

Compared to the fluoxetine group, the CLz/F of fluoxetine in multiple dose group C was significantly decreased and the MRT (0–*t*) was significantly increased. Compared to the fluoxetine group, the CLz/F of NOF in multiple dose groups A and B were significantly increased, and the MRT (0–*t*) of NOF in multiple dose group C was significantly decreased.

There was no significant difference in t1/2z of fluoxetine and norfluoxetine among each group. The *T*_max_ values of fluoxetine in the fluoxetine group, multiple dose group A, multiple dose group B, and multiple dose group C were 0.75, 1, 1, and 1 h, respectively, and the *C*_max_ values were 2.19, 1.58, 2.14, and 2.38 *μ*g/L, respectively. The *T*_max_ values of norfluoxetine were 2, 2, 1, and 1 h, respectively, and the *C*_max_ values were 8.19, 3.97, 4.64, and 16.62 *μ*g/L, respectively.

Blood concentration method is a classical pharmacokinetic research method, which can clearly express drug absorption, distribution, bioavailability, biotransformation, and excretion [22]. In this study, an LC-MS/MS method for the estimation of FLU and NOF in plasma was developed to assess potential effects of CSGS on pharmacokinetics of FLU.

The *T*_max_ of fluoxetine in each group was close, but the *C*_max_ of fluoxetine in multiple dose group A decreased by 29% compared with the fluoxetine group, while multiple dose group C increased by 9%. Similarly, compared with the fluoxetine group, the AUC (0–∞) of fluoxetine in the multiple dose group C increased by 80%, while multiple dose groups A and B decreased by 96% and 51%, respectively. All above results suggested that high dose of CSGS can promote the absorption of fluoxetine, while the low dose of CSGS can inhibit it.

Besides, the CLz/F of fluoxetine in the multiple dose group C was lower than that of fluoxetine group, indicating that high dose of CSGS can inhibit the excretion of fluoxetine.

The *C*_max_ of norfluoxetine in multiple dose groups A and B decreased by 52% and 43%, respectively, while the multiple dose group C increased by 103%. Meanwhile, the AUC (0–∞) of norfluoxetine in the multiple dose group C was higher than that of the fluoxetine group with the AUC (0–∞) of norfluoxetine in multiple dose group A and B being lower. The above results suggested that low and middle dose of CSGS can inhibit metabolism of fluoxetine to norfluoxetine, while high dose of CSGS can promote it. Although there was no significant difference in CLz/F between the fluoxetine group and multiple dose group C, the CLz/F of norfluoxetine in multiple dose group A and B was higher than that of fluoxetine group, indicating that low dose and middle dose of CSGS can promote the elimination of norfluoxetine. As shown in Figure 3, the concentration of norfluoxetine in each group increased again after declining but showed a high standard deviation. Although we have done a lot of work to minimize the standard deviation, a large interindividual variability exists in the plasma concentrations of FLU after administration of the same dose of the drug, which may be partly related to the activity of CYP2C9 [23]. We will increase the sample size and further study its possible mechanism in the next study.

This study indicated that the high dose of CSGS may promote the absorption and metabolism of fluoxetine to norfluoxetine, while low dose and middle dose of CSGS may inhibit the absorption and metabolism of fluoxetine to norfluoxetine and inhibit elimination of norfluoxetine. Gastrointestinal motility has a huge impact on pharmacokinetics of orally administered drugs [24]. Studies have shown that the bioavailability of cyclosporin A (CyA) is reduced after oral administration of the gastrointestinal motility inhibitor atropine. As the dose of atropine increased, the area under the concentration-time curve (AUC) and the peak blood concentration of CyA decreased [25]. Drugs used to promote gastrointestinal motility can alter the pharmacokinetics of some coadministered drugs [26]. Fructus Aurantii flavonoids are one of the main components of Fructus Aurantii that possess prominent gastrointestinal motility promoting efficacy. Fructus Aurantii flavonoids can regulate the content of 4-dimethylallyltryptophan, corticosterone, phytosphingosine, sphinganine, and LysoPC through tryptophan metabolism, corticosterone metabolism, sphingolipid metabolism, and other pathways to present its gastrointestinal motility promoting efficacy [27]. Previous studies have shown that Aurantii Fructus immaturus flavonoid (AFIF) could improve the contraction of isolated gastric smooth muscle strips in rats, which had a diastolic effect on PCSMS. This effect is closely related to NOS activation, cGMP and PKG upregulation, and decrease of intracellular Ca2+ concentrations in smooth muscle [28]. Studies on the potential effects of meranzin hydrate (MH) and decoction of herb Fructus Aurantii (FA) on rat gut motility have found that low-dose FA fails to accelerate intestinal transport and gastric emptying, while high-dose FA promotes rat gut motility [29]. Meranzin hydrate (MH) and Fructus Aurantii flavonoids may be the main pharmacodynamic substance of CSGS, and different doses of CSGS may have different effects on gastrointestinal function.

On the other hand, FLU is mainly metabolized to NOF by 3A4 isozyme of cytochrome P-450 (CYP3A4) in liver [30–32]. Meanwhile, FLU and NOF have been shown to be potent inhibitors of CYP3A and CYP2D6 and thus interact with many other drugs that are metabolizing through this enzyme [33, 34]. FLU and its circulating metabolite, NOF, contain a complex multiple inhibitor system that causes reversible or time-dependent inhibition of cytochrome P-450 (CYP) family members, CYP2D6, CYP3A4, and CYP2C19. Continuous administration of FLU for 2 weeks did not affect the AUCs of midazolam and lovastatin but increased the AUC of dextromethorphan and omeprazole 27 times and 7.1 times, respectively [35].

CSGS consists of seven Chinese herbs: the root of *Bupleurum chinense* DC. (Chai-Hu), the root of *Paeonia lactiflora* Pall. (Bai-Shao), the pericarps of *Citrus reticulata* Blanco (Chen-Pi), the root of *Ligusticum chuanxiong* Hort. (Chuan-Xiong), the root of *Cyperus rotundus* L. (Xiang-Fu), the fruit of *Citrus aurantium* L. (Zhi-Qiao), and the root of *Glycyrrhiza uralensis* Fisch. (Gan-Cao) [36, 37]. In these herbs, most components have been identified by LC-MS/MS, including ferulic acid, albiflorin, glycyrrhizic acid, hesperidin, glycyrrhetic acid, liquiritin, isoliquiritigenin, neohesperidin, merazin hydrate, paeoniflorin, and naringin [38–41], which mainly contain macromolecular substances such as saponins and flavonoids. Fructus Aurantii is close to grapefruit from the perspective of botanical taxonomy or components, and their main ingredients are flavonoids. Grapefruit has been proven to cause drug-drug interaction when coadministrated with CYP3A4 substrates [42, 43]. Flavonoids inhibit OATP1B1- and OATP1B3-mediated drug delivery [44]. Water extract of FA immature increased CYP3A4 protein expression and ethanol extract of FA immature induced CYP3A4 expression via the induction of PXR expression [45]. FA may be a potential minor inducer of CYP1A2 and CYP3A4 [46]. Related research shows that resveratrol may interfere with albumin binding of site II ligands and metabolism of drugs by CYP2C9 and/or CYP3A4 enzymes [47]. Resveratrol (Res) enhances methotrexate (MTX) absorption in the intestine and reduces MTX kidney elimination by inhibiting P-gp, MRP2, OAT1, and OAT3 in vivo and in vitro. Res improves MTX-induced kidney damage without increasing intestinal toxicity [48]. Resveratrol significantly increased the AUC and *C*_max_ of aripiprazole (APZ) by inhibiting CYP3A4 and CYP2D6 enzymes [49]. Pretreatment with naringin may significantly increase plasma concentration-time curve (AUC), drug peak (*C*_max_), absolute bioavailability (AB%), and relative bioavailability (RB%) of tamoxifen and its metabolite 4-hydroxy tamoxifen by inhibiting CYP3A4 enzyme [50]. The macromolecular components of glycyrrhizin, such as saponins and flavonoids, affect drug solubility, permeability, distribution, and metabolism. Glycyrrhizin has been shown to alter the enzymatic activity of P-450 isoforms by inducing model probe substrates, as well as to regulate drug transporters such as intestinal P-glycoprotein, which ultimately affects drug metabolism. [51, 52] When *Glycyrrhiza uralensis* was combined with lidocaine, *Glycyrrhiza uralensis* reduced the half-life of lidocaine by 39% and the total clearance by 59% by inducing P450 isoenzyme [53]. Liquorice extract (LE) and its main component glycyrrhizin (GZ) combined with cyclosporin (CsA) significantly reduced the peak blood concentration of CsA in the blood of rats and the area under the curve of CsA; licorice extract (LE) may significantly reduce the oral bioavailability of CsA by activating P-gp and CYP3A4 [54]. Glycyrrhizin (GZ) and licorice significantly increased the AUC and MRT of methotrexate (MTX) [55].

Mu et al. [56] found that *Glycyrrhiza* can induce CYP3A and CYP2C expression by activating PXR to promote the metabolism of warfarin in rats. Sun et al. [57] found that *Glycyrrhiza* could increase the absorption of substrate rhodamine 123 (R123) by inhibiting P-gp activity. However, studies on the pharmacokinetics interactions between rosuvastatin and naringin in rats showed that naringin has no effect on the drug metabolism of rosuvastatin [58]. By contrary, it is reported that hesperidin and naringenin could reduce the activity of CYP3A4 and P-gp to inhibit metabolism of felodipine [59, 60].

The results of this study suggested that more than one enzyme can be involved in this complex process, and the underlying mechanism needs further investigation. However, we could speculate that this may be related to the quantity of active principle absorption from different doses of CSGS. When high dose of CSGS is taken, the enzymes like CYP3A4, CYP2D6, and P-gp may be inhibited, whereas when low dose and middle dose of CSGS are taken, the opposite results may be obtained.

## 3. Materials and Methods

### 3.1. Reagents and Materials

The reference standards of fluoxetine, norfluoxetine, and sulfamethoxazole (SMZ) were obtained from China National Institute for Drug and Biological Products. HPLC-grade methanol and formic acid were purchased from Sigma company (USA). Ultrapure water was purchased from Wahaha Group Co. Ltd. (Hangzhou, China). Ethyl acetate and ammonium chloride buffer solution (pH 10) were purchased from Hengxing Chemical Reagent Co. Ltd. (Tianjin, China).

### 3.2. Raw Herbal Medicines and Western Medicine

All raw herbal medicines, Chai-Hu, Chen-Pi, Bai-Shao, Zhi-Qiao, Xiang-Fu, Chuan-Xiong, and Gan-Cao, were purchased from Xiangya Hospital Pharmacy (Hunan, China). The original herbal ingredients of CSGS were mixed and crushed into small pieces in a ratio of 4 : 4 : 3 : 3 : 3 : 3 : 1. The compound was immersed in water (1 : 8, w/v) for 30 minutes at room temperature, then heated to boiling, and boiled for a further 0.5 hours. The filtrate was collected, and the residue was refluxed in the same volume of water and heated for a further 0.5 hour. The two filtrates were combined and concentrated in vacuo to give a CSGS extract of 2.1 g/ml. When used, it is made into a certain concentration with distilled water as needed.

Fluoxetine hydrochloride, bought from Lilly Pharmaceutical Co. Ltd. was dissolved in pure water at the concentration 0.18 mg/ml.

### 3.3. Experimental Animal and Drug Administration

Thirty-two healthy adult male Sprague-Dawley (SD) rats, weighing 200 ± 20 g, were purchased from Experimental Animal Science of Xiangya Medical College of Central South University (SYXK (Xiang) 2016-0761). The rats were housed individually, lit at 07 : 00 am. The feeding space is maintained at an ambient temperature of 23–25°C and a relative humidity of 54%–66%. Throughout the experiment, animals were given food and water unless otherwise stated. All procedures were approved and implemented in accordance with the guidelines of the ethics of Xiangya Hospital in Central South University.

Thirty-two SD rats were randomly divided into four groups: the fluoxetine group and multiple dose groups A, B, and C, and different groups were orally administered with a combination of FLU and different doses of CSGS for 14 d. The rats in the fluoxetine group were administered FLU (1.8 mg/kg·d). The rats in multiple dose group A were administered a combination of FLU and low dose of CSGS (CSGS 5.9 g/kg·d, FLU 1.8 mg/kg·d). The rats in multiple dose group B were administered a combination of FLU and middle dose of CSGS (CSGS 11.8 g/kg·d, FLU 1.8 mg/kg·d). The rats in multiple dose group C were administered a combination of FLU and high dose of CSGS (CSGS 23.6 g/kg·d, FLU 1.8 mg/kg·d). In the fifteenth day, serial blood samples were taken from the caudal vein before the administration and at 0.25, 0.5, 0.75, 1, 2, 4, 6, 8, 10, 12, 24, 36, and 48 h after the administration. And, instantly, the blood sample was centrifuged at 3500 rpm for 15 min (4°C) and the supernatant was gathered as the plasma and stored at −20°C before analysis.

### 3.4. Sample Preparation

A liquid-liquid extraction method was applied to extract the analytes from serum.

Before analysis, the plasma samples were thawed to room temperature. An aliquot of 100 *μ*l plasma samples were placed in 1.5 mL Eppendorf tubes. Then, 40 *μ*l ammonium chloride buffer solution (pH 10), 50 *μ*l methanol, and 50 *μ*l IS were added into each tube. The mixture was vortex mixed for 30 s and mixed with 1 ml ethyl acetate. Next, an ultrasonic bath was applied for 30 min, and the mixture was centrifuged for 15 min at 12000 revolutions per minute. The supernatant was collected and transferred into a 1.5 mL Eppendorf tube and then dried under nitrogen stream at 40°C. The residue was dissolved with 600 *μ*l of 50% methanol, and then, the solution was centrifuged at 12,000 rpm for 15 min. Finally, 5 *μ*l of supernatant of the solution was injected in the LC-MS/MS for analysis.

### 3.5. Preparation of Calibration Standard and Quality Control (QC) Samples

Fluoxetine and norfluoxetine were dissolved in methanol to produce a standard solution (520 *μ*g/ml and 1000 *μ*g/ml) and then diluted in Eppendorf vials to make a stock solution (384 *μ*g/mL). Subsequently, stock solutions were strictly diluted with methanol solution to provide nine standard working solutions for fluoxetine: 192, 96, 48, 24, 12, 4, 1.333, 0.444, and 0.111 ng/ml and norfluoxetine: 384, 192, 96, 48, 24, 8, 2.667, 0.889, and 0.222 ng/ml. SMZ (IS) was dissolved in methanol to make a stock solution (130 *μ*g/Ml).

Quality control (QC) samples were prepared in the same way as mentioned above. Fluoxetine and norfluoxetine stock solutions were strictly diluted with methanol solution to provide three standard working solutions for fluoxetine: 1, 4, and 16 ng/ml and norfluoxetine: 2, 4, and 8 ng/ml.

All these solutions were stored at 4°C and brought to room temperature before the solutions were used.

### 3.6. Instrumentation and LC-MS/MS Conditions

Liquid chromatography was performed on an Agilent 1290 series liquid chromatography system (Palo Alto, CA, USA), with an Zorbax Eclipse C18 column (4.6 mm × 250 mm, 3.5 *μ*m) maintained at 40°C temperature at a flow rate of 0.2 mL/min. The mobile phases consisted of methanol (A) and 1% formic acid (B) with a gradient elution of 40% A at 0–5 min; 40%–50% A at 5–10 min; and 50%–60% A at 10–20 min. The total run time was 20.0 min. The injection volume was 5 *μ*l in the partial loop mode, and the temperature of the autosampler was set at 25°C.

The detection was performed on an API 3200 triple quadrupole mass spectrometer with an electrospray ionization (ESI) source, and the mass spectrometer was operated in multiple reaction monitoring (MRM) mode. The MRM transitions for the fluoxetine, norfluoxetine, and IS were *m*/*z* 310 ⟶ 148.1 (collision energy of 20 eV, fragmentation voltage of 65 V), *m*/*z* 296 ⟶ 134.1 (collision energy of 20 eV, fragmentation voltage of 65 V), and *m*/*z* 254.100 ⟶ 108.100 (collision energy of 22 eV, fragmentation voltage of 50 V), respectively. The source parameters were set as follows: capillary voltage was 5.5 kV, gas temperature was 500°C; nebulizer gas (N2) was 50 psig; curtain gas was 20 psig; and turbo gas was 5 psig. Agilent Quantitative analysis software was used for data analysis.

### 3.7. Method Validation

The method described in this study was validated for selectivity, linearity, precision, accuracy, recovery, and stability according to the CFDA [61]. According to the CFDA guidance for bioanalytical method validation: when the content of tested components is less than 1 mg/L, the permissible limit of recovery is 80%–120%; when the content of tested components is 1–100 mg/L, the permissible limit of recovery is 90%–110%; when the content of tested components is more than 100 mg/L, the permissible limit of recovery is 95%–105%.

#### 3.7.1. Linearity and Sensitivity

Calibration curves were generated by spiking blank rat plasma with different concentrations of the working solutions. The ratios of analyte to IS area versus analyte concentration were used for regression analysis. Each calibration curve was analyzed individually by using least square weighted (1/*x*^2^) linear regression. The linearity of the assay was assessed using the coefficient of determination (*r*^2^) for the calibration curve, which should be greater than 0.995.

#### 3.7.2. Specificity

The specificity was used to check whether endogenous substances interfered with analytes and IS or not. The specificity was evaluated by comparing the chromatograms of analytes-free plasma containing neither analytes nor IS (double blank) with those of corresponding spiked plasma and real plasma sample after administration.

#### 3.7.3. Precision and Accuracy

Intraday and interday precisions were done by repeating the analysis at low, medium, and high concentration levels in five separate runs on the same day (intraday precision) and on three consecutive days (interday precision). The relative standard deviations (RSDs) were used to evaluate the intraday and interday variations. The RSD was expected to be within ±15.0%.

#### 3.7.4. Stability

The stability tests were designed to cover the anticipated conditions that the samples might be exposed during storage and handling, including reanalyzing QC samples at room temperature for 4 h, three freeze-thaw cycles, at the storage temperature (−20°C) for 4 weeks. All stability studies were evaluated at low, medium, and high QC levels with five determinations for each test. The relative standard deviations (RSDs) were used to evaluate the stability of the analytes. The RSD was expected to be within ±15.0%.

#### 3.7.5. Recovery and Matrix Effect

The recovery of the analytes and IS was calculated by comparing peak areas of extracted plasma samples with postextracted spiked samples at the same theoretical concentrations at three concentrations (low, medium, and high) with five determinations. The matrix effect was assessed by comparing the peak area of the analytes in postextracted spiked samples with standard solutions at the same theoretical concentrations at three concentrations (low, medium, and high) with five determinations.

### 3.8. Pharmacokinetic Analysis

All parameters, including the area under the plasma concentration-time curve (AUC), maximal plasma concentration (*C*_max_), time of the maximal plasma concentration (*T*_max_), the mean residence time (MRT), drug half-life (*T*1/2), clearance (CL), were calculated using Drug and Statistics 3.2.8 (DAS 3.2.8, Mathematical Pharmacology Professional Committee of China, Shanghai, China).

### 3.9. Statistical Analysis

The results were reported as the mean ± standard error of the mean (SEM). Statistical analysis was performed by one-way ANOVA using SPSS 19.2 software. A *P* value <0.05 showed statistical significance.

## 4. Conclusions

In order to assess potential effects of CSGS on pharmacokinetics of fluoxetine, an LC-MS/MS method was developed. There were some differences in pharmacokinetic parameters between the FLU group and multiple dose groups, and CSGS can affect the pharmacokinetics of fluoxetine.

## Figures and Tables

**Table 1:** Regression data of the analytes determined.

Composition	Regression equation	*R* ^2^
FLU	*Y* = 15.452*x* − 0.0385	0.99742
NOF	*Y* = 8.2126*x* + 0.2367	0.99875

FLU: fluoxetine; NOF: norfluoxetine.

**Table 2:** Intraday and interday precision of the analytes (*n* = 5).

Composition	Concentration (ng/ml)	Intraday precision		Interday precision	
		Measured (ng/ml)	RSD%	Measured (ng/ml)	RSD%
FLU	1	0.980 ± 0.023	2.35	0.964 ± 0.026	2.70
	4	3.974 ± 0.197	4.96	3.958 ± 0.187	4.72
	16	15.922 ± 0.745	4.68	15.836 ± 0.815	5.15

NOF	2	1.932 ± 0.064	3.31	1.884 ± 0.056	3.00
	4	3.931 ± 0.147	3.74	3.941 ± 0.135	3.42
	8	7.911 ± 0.332	4.20	7.881 ± 0.342	4.34

FLU: fluoxetine; NOF: norfluoxetine.

**Table 3:** Stability of the analytes (*n* = 5).

	Concentration (ng/ml)	At room temperature for 4 h		Freeze-thaw three cycles		−20°C for 4 weeks	
		Measured *C* (ng/ml)	RSD%	Measured *C* (ng/ml)	RSD%	Measured *C* (ng/ml)	RSD%
FLU	1	1.010 ± 0.028	2.77	0.997 ± 0.012	1.20	0.987 ± 0.018	1.82
	4	3.965 ± 0.210	5.30	3.991 ± 0.212	5.31	4.012 ± 0.231	5.76
	16	15.832 ± 0.536	3.39	15.872 ± 0.845	5.32	15.872 ± 0.654	4.12

NOF	2	1.943 ± 0.054	2.78	1.879 ± 0.045	2.39	1.873 ± 0.044	2.35
	4	3.934 ± 0.186	4.73	4.012 ± 0.231	5.76	3.891 ± 0.156	4.01
	8	7.897 ± 0.234	2.96	7.987 ± 0.341	4.27	7.871 ± 0.246	3.13

FLU: fluoxetine; NOF: norfluoxetine.

**Table 4:** Recovery of analytes (*n* = 5).

Composition	Concentration (ng/ml)	Measured *C* (ng/ml)	Recovery (%)	RSD%
FLU	1	1.040 ± 0.023	104.06	2.19
	4	4.046 ± 0.257	101.16	6.36
	16	16.322 ± 0.765	102.01	4.68

NOF	2	1.846 ± 0.074	92.31	3.99
	4	3.831 ± 0.238	95.78	6.20
	8	7.901 ± 0.436	98.88	5.52

FLU: fluoxetine; NOF: norfluoxetine.

**Table 5:** Mean matrix effect of analytes (*n* = 5).

Composition	Concentration (ng/ml)	Measured *C* (ng/ml)	Matrix effect (%)	RSD%
FLU	1	1.015 ± 0.027	101.03	2.67
	4	4.011 ± 0.149	100.28	3.71
	16	16.029 ± 0.676	100.18	4.21

NOF	2	1.850 ± 0.079	92.53	4.27
	4	3.834 ± 0.197	95.85	5.13
	8	7.825 ± 0.378	97.81	4.83

FLU: fluoxetine; NOF: norfluoxetine.

**Table 6:** Pharmacokinetic parameters of fluoxetine.

Parameter	Unit	Fluoxetine group	Multiple dose group A	Multiple dose group B	Multiple dose group C
AUC (0–∞)	ug/L∗h	12.20 ± 2.67	11.20 ± 3.86	14.54 ± 3.22	22.01 ± 4.49^a^
MRT (0–*t*)	h	7.67 ± 1.02	6.82 ± 0.77	6.88 ± 0.74	10.12 ± 1.11^a^
t1/2z	h	8.95 ± 7.50	5.46 ± 0.25	4.93 ± 0.34	8.34 ± 3.31
*T*_max_	h	0.75	1	1	1
CLz/F	L/h/kg	149.29 ± 28.93	178.71 ± 55.98	130.48 ± 31.64	82.49 ± 12.18^a^
*C*_max_	ug/L	2.19	1.58	2.14	2.38

Fluoxetine group: fluoxetine 1.8 mg/kg·d for consecutive 14 d; multiple dose group A: CSGS 5.9 g/kg·d and fluoxetine 1.8 mg/kg·d for consecutive 14 d; multiple dose group B: CSGS 11.8 g/kg·d and fluoxetine 1.8 mg/kg·d for consecutive 14 d; multiple dose group C: CSGS 23.6 g/kg·d and fluoxetine 1.8 mg/kg·d for consecutive 14 d. ^a^*P* < 0.05, significantly different from the fluoxetine group.

**Table 7:** Pharmacokinetic parameters of norfluoxetine.

Parameter	Unit	Fluoxetine group	Multiple dose group A	Multiple dose group B	Multiple dose group C
AUC (0–∞)	Ug/L∗h	181.93 ± 45.71	53.27 ± 11.90^a^	101.74 ± 24.46	284.02 ± 49.07^a^
MRT (0–*t*)	h	15.94 ± 1.22	16.15 ± 1.42	17.53 ± 2.13	12.71 ± 0.68^a^
t1/2z	h	13.76 ± 5.34	20.24 ± 9.96	9.31 ± 2.28	10.79 ± 2.52
*T*_max_	h	2	2	1	1
CLz/F	L/h/kg	9.47 ± 2.16	29.63 ± 4.88^a^	17.81 ± 4.63^a^	6.21 ± 1.07
*C*_max_	ug/L	8.19	3.97	4.64	16.62

Fluoxetine group: fluoxetine 1.8 mg/kg·d for consecutive 14 d; multiple dose group A: CSGS 5.9 g/kg·d and fluoxetine 1.8 mg/kg·d for consecutive 14 d; multiple dose group B: CSGS 11.8 g/kg·d and fluoxetine 1.8 mg/kg·d for consecutive 14 d; multiple dose group C: CSGS 23.6 g/kg·d and fluoxetine 1.8 mg/kg·d for consecutive 14 d. ^a^*P* < 0.05, significantly different from the fluoxetine group.

## Data Availability

The data used to support the findings of this study are available from the corresponding author upon request.
